# Preparing Students for the Public Health Workforce: The Role of Effective High-Impact Educational Practices in Undergraduate Public Health Program Curricula

**DOI:** 10.3389/fpubh.2022.790406

**Published:** 2022-03-24

**Authors:** Elizabeth A. Armstrong-Mensah, Kim Ramsey-White, Ernest Alema-Mensah, Barbara A. Yankey

**Affiliations:** ^1^Georgia State University, Department of Health Policy and Behavioral Sciences, Atlanta, GA, United States; ^2^Morehouse School of Medicine, Atlanta, GA, United States

**Keywords:** high-impact educational practices, public health workforce, undergraduate public health curriculum, study abroad, signature experience, undergraduate research

## Abstract

There are several institutions of higher learning in the United States that award degrees in public health to undergraduate students. While these institutions serve as potential pipelines for the public health workforce, it is unclear if the curricula and training students receive from these institutions, really prepare them for the public health workforce or higher education. The questions sometimes asked are whether the programs offered by these institutions exist to provide students with a good understanding of public health issues so they can become good citizens for building a responsible society, or if it is to prepare students for graduate school. Regardless of what the goals are, students in undergraduate public health programs need to be exposed to curricula that adequately prepare them to enter well-defined careers in public health. Thus, institutions of higher learning offering degrees in public health to undergraduate students need to understand the market, assess, and understand the needs of public health agencies, and tailor course curricula to match those needs. Georgia State University established its undergraduate public health program in 2016. Since then, over 200 students have graduated from the program. The purpose of the study was to assess student perception of the role of high impact educational practices such as study abroad, signature experience, and undergraduate research curricula in preparing them for careers in public health.

## Introduction

High-impact educational practices (HIPs) are practices that promote deep learning through student engagement. They focus on the knowledge, attitudes, and skills college students need to succeed academically and professionally. HIPs have the ability to transform students' personal development and educational growth, as well as to improve the quality of students' experience, learning, retention, and success (1). These educational practices also afford students the opportunity to participate in activities beyond the classroom, resulting in learning and personal development (2). HIPs not only enable students to apply what they have learned; they also contribute to metacognitive gains by students (3).

HIPs take various forms and include study abroad, signature experience, first year seminars, learning communities, writing intensive courses, collaborative research, and undergraduate research, all of which have been widely tested and found to be beneficial to the cumulative learning of college students enrolled in all types of programs (4, 5). HIPs are powerful educational practices in that, they require applied, hands-on, integrative, and often collaborative learning experiences. Georgia State University (GSU), a large, urban, public research institution located in the heart of Atlanta, Georgia, serves over 400 undergraduate students in the Bachelor of Science in Public Health (BSPH) program. At GSU, students across the university including those within the School of Public Health (SPH) have access to a variety of HIPs opportunities including study abroad, signature experience, and undergraduate research.

Opportunities to study abroad continue to be a popular choice for college students in the United States (US) looking to expand their undergraduate education. Per figures released by the Open Doors Report on International Educational Exchange, about 273,996 US students studied abroad for credit during the 2010-2011 academic year (6). Participating in such opportunities have helped to develop students' global and intercultural competencies (7). Corroborating this fact, Braskamp et al. (8) found that education abroad increases student intercultural competence, intercultural maturity, and intercultural sensitivity. Signature experience courses make learning come alive and provide real-world engagement with course content for students.

Public health signature experiences give public health majors the opportunity to integrate, synthesize and apply their public health knowledge through cumulative and experiential activities. As part of the course offering, students usually complete a variety of projects, and written assignments designed to assess their acquisition of the required public health competencies covered within the public health major. The course encompasses a holistic review of the field of public health while integrating student reflection on what they have learned and how they may apply their knowledge to future educational and career aspirations.

The GSU BSPH program is a generalist degree with a focus on urban and global health. The interdisciplinary makeup of the faculty and the diverse research projects currently underway in the school presents fertile ground for students to have diverse, experiential learning opportunities. The GSU BSPH program seeks to prepare students for work across public health disciplines, and to equip graduates with cross-professional competencies for public health jobs with local and global public health organizations. While GSU BSPH programs serve as a pipeline for the public health workforce, the question is whether the curricula of the courses offered actually prepare students to competently enter the public health workforce or to pursue graduate education. To date, no study has been conducted to assess the perception of students on whether the HIPs associated with GSU BSPH program curricula adequately prepares them for careers in public health. Thus, the purpose of the study was to assess student perceptions of the role of HIPs such as study abroad, signature experience, and undergraduate research curricula, in preparing students for careers in public health.

## Materials and Methods

### Setting and Population

The GSU SPH offers six-degree programs (Bachelor of Science in Public Health; Master of Public Health; Graduate Certificate in Public Health; Graduate Certificate in Maternal and Child Health; Doctor of Philosophy; and the Doctor of Public Health) and serves over 700 students across all programs. The student body comprises traditional and non-traditional students from diverse racial and socio-economic backgrounds. The GSU BSPH program seeks to equip students with interdisciplinary understanding of public health, using a variety of approaches and course work. Consequently, students enrolled in these programs gain knowledge and acquire skills needed for graduate school, and careers in a wide range of public health and interdisciplinary professions.

### Sampling and Data Collection

Using purposive sampling, we conducted a cross-sectional study to assess GSU BSPH student perceptions of the role of HIPs in preparing them for careers in public health. Students enrolled in the program during the summer and spring of 2021 were informed about and invited to participate in the study through faculty announcements, emails, and information posted on iCollege, the university's learning management system. Students were informed that the study was voluntary and that they could decide not to participate. Students who volunteered to participate in the study indicated their consent to participate by completing an electronic questionnaire created in Qualtrics. To uphold student confidentiality and privacy, no personally identifiable information was collected. The GSU institutional review board (IRB) determined that the study was exempt from federal regulation.

### Variables and Measurement

The data collection instrument comprised 15 multiple choice and closed ended questions across five domains – i) demographic and academic information. ii) GSU BSPH program curricula, iii) study abroad, iv) signature experience, and v) undergraduate research. The demographic and academic information domain questions focused on student academic status, gender, age, and race. The GSU BSPH program curricula domain questions focused on whether the program curricula prepare students for jobs in public health. The study abroad domain questions focused on whether GSU study abroad programs are designed in a way that help students to develop global fluency skills for future careers in public health. The signature experience domain included questions on whether the GSU BSPH signature experience course prepares students to be successful at interviews. It also focused on whether students are able to articulate the knowledge and skills they have acquired from the program, and whether students thought the program equipped them with the competencies required for entry level public health jobs. The undergraduate research questions focused on student perceptions of the relevance of research for professional advancement, whether the GSU BSPH program gives undergraduate students research opportunities, and the research skills students possess.

### Statistical Analysis

Collected data in Qualtrics was cleaned and exported to SAS version 9.4 for analysis. Descriptive, bivariate, and multivariate logistic regression analysis were conducted at a confidence level of alpha = 0.05. Missing quantitative data were excluded from calculations. In cases where “No” responses were below 5, and thus, too small to be analyzed alone, they were combined with “Do not know” responses [shown in the relevant rows in the multivariate (Table 1)]. One response registered as unknown for gender was dropped in the multivariate analysis, since this response could not be analyzed alone or combined with either of the two responses for the gender category. Descriptive analysis was conducted to summarize data. Multivariate analysis was conducted to assess the relationship between whether GSU BSPH curricula including associated factors (study abroad, signature experience, and undergraduate research) prepares students for jobs in public health.

**Table 1:** Multivariate analysis of GSU BSPH program curricula prepares students for jobs and other BSPH curricula associated factors (study abroad, signature experience and undergraduate research).

**Variables**	**Odds ratio**	***P*-value**	**95% Confidence interval**
**Undergraduate research**			
Would you say that the GSU BSPH program provides students with research opportunities? Yes/I do not know	6.76	0.035	1.15–39.82
**Study abroad**			
Would you say that GSU study abroad programs are designed in a way that helps students to develop global fluency skills for future careers in public health? Yes/Have not participated	1.71	0.242	0.70–4.23
**Signature experience**			
Would you say that the GSU signature experience course teaches students how to articulate the knowledge and skills they have acquired from the program? Yes/I do not know	0.95	0.924	0.37–2.69
**GSU BSPH program curricula**			
Would you say that the GSU BSPH program curriculum prepares students for graduate education? Yes/No	30.83	0.001	4.27–222.47
**Demographics**			
Gender: male vs. female	0.27	0.235	0.03–2.32
Academic status: junior to senior	1.20	0.735	0.43–3.36
Age, 18–24 yrs vs. 25+	0.15	0.094	0.02–1.38
African American vs. all other	0.90	0.615	0.59–1.37

## Results

### Univariate Statistics

#### Demographic and Academic Information

A total of the 64 students enrolled in the GSU BSPH program participated in the survey. Of this number, 1.6% was a freshman, 15.5% were sophomores, 34.4% were juniors, and 48.4% were seniors. The majority of students (78.1%) self-identified as female and were within the 18–24 years age range (71.9%) (Table 2).

**Table 2:** Descriptive characteristics of participants by five identified domains.

**Variables**	**Sample size, *n***	**Percentage, %**
**1. Demographics and academic information**		
**Academic status**		
Freshman	1	1.6
Sophomore	10	15.6
Junior	22	34.4
Senior	31	48.4
Total	64	100.0
**Age**		
18–24 years	46	71.9
25–30 years	5	7.8
30+	13	20.3
Total	64	100.0
**Gender**		
Male	13	20.3
Female	50	78.1
Other	1	1.6
Total	64	100.0
**2. GSU BSPH program curricula**		
**GSU BSPH curricula prepares students for jobs**		
Yes	38	61.3
No	5	8.1
I do not know	19	30.6
Total	62	100
**GSU BSPH curricula prepares students for graduate education**		
Yes	42	68.9
No	1	1.6
I do not know	18	29.5
Total	61	100.0
**3. Study abroad**		
**GSU study abroad programs help students develop global fluency skills for careers in public health**	20	33.3
Yes		
No	-	-
I have not participated in study abroad	40	66.7
Total	60	100.0
**GSU study abroad programs equip students with careers in public health (Multiple)**		
Empathy	6	8.3
Cultural sensitivity	13	18.1
Respect for others	13	18.1
Willingness to serve	11	15.3
I have not participated in study abroad	48	66.7
**4. Signature experience**		
**GSU signature experience course designed to helps students demonstrate behaviors that will help them perform well in real-world job situations**		
Yes	15	26.8
No	-	-
I have not participated in signature experience	41	73.2
Total	56	100.0
**Competencies required for entry level public health careers that GSU signature experience course helps students acquire (Multiple)**		
Analysis of public health issues	10	13.9
Critical thinking	13	18.1
Interpersonal skills	9	12.5
Problem solving	9	12.5
Leadership skills	9	12.5
Communication of public health issues	10	13.9
I have not participated in signature experience	39	54.2
**The GSU signature experience course prepares students for job interviews** Yes		
Yes	10	18.2
No	1	1.8
I do not know	44	80.0
Total	55	100
**GSU signature experience course teaches students how to articulate the knowledge and skills they have acquired from the program**		
Yes	17	30.4
No	-	-
I do not know	39	69.6
Total	56	100
**5. Undergraduate reserach**		
**GSU BSPH program provides students with research opportunities** Yes	40	71.4
No	5	8.9
I do not know	11	19.6
Total	56	100
**Participating in undergraduate research is relevant for professional advancement**		
Yes	50	89.3
No	3	5.4
I do not know	3	5.4
Total	56	100
**Which research skills do you have (Multiple)**		
Literature review	33	45.8
Annotated bibliography	37	51.4
Data collection	33	45.8
Data analysis	37	51.4
Academic writing	40	55.6
Critical thinking	47	65.3
Doing citations	42	58.3
None	3	4.2

#### GSU BSPH Program Curricula

Concerning the GSU BSPH program curricula and whether it prepares undergraduates for public health jobs, 38% of students stated that they did (“yes”), 8.1% thought otherwise (“no”), and 30.6% said they did not know. The majority of participants (68.9%) stated that the GSU BSPH program curricula prepares students for graduate education. Not as many students (1.6%) thought otherwise (Table 2).

#### Study Abroad

As shown in Table 2, regarding whether GSU study abroad programs help students develop global fluency skills for careers in public health, 33.3% of students answered in the affirmative (“yes”), while 66.7% of students did not take a stand as they said they had not yet participated in any GSU study abroad program. No student indicated that the programs were not helpful. Most students (18.1%) indicated that GSU study abroad programs help to equip students with cultural sensitivity skills and teaches students how to be respectful of people from other cultures (18.1%). According to 15.3% of students, GSU study abroad programs create a “willingness to serve” attitude among students (Table 2).

#### Signature Experience

A little over a fourth of students (26.8%) indicated that the GSU signature experience course helps students to demonstrate behaviors that will help them perform well in real job situations globally and locally. 73.2% of the students said they did not know as they were not yet seniors. Regarding competencies required for entry level public health jobs, some students (13.9%) said that the course equips students with the ability to analyze public health issues, think critically (18.1%), develop interpersonal skills (12.5%), solve problems (13.9%), acquire leadership skills (13.9%), and to communicate public health issues (13.9%). Over half of the students (54.2%) did not have an opinion on the matter as they had not yet taken the signature experience course. In response to whether the GSU signature experience course prepares students for job interviews, 18% of students said “yes” and 1.8% said “no”. Some students (80%) stated that they did not know. About a third of students (30.4%) reported that the GSU signature experience course helps students articulate their public health knowledge and skills (Table 2).

#### Undergraduate Research

As presented in Table 2, 71.4% of students who participated in the study indicated that the GSU BSPH program provides opportunities for undergraduate research compared to 8.9 % who did not think so. Per the majority of students (89.3%), participating in undergraduate research is relevant for professional advancement. Some research skills students listed they have as a result of participating in undergraduate research are doing literature review (45.8%), annotated bibliography (51.4%), data collection (45.8%), data analysis (51.4%), academic writing (55.6%), critical thinking (65.3%), and doing citations (58.3%) (Table 2).

### Bivariate and Multivariate Analysis

We conducted bivariate analysis to examine the relationship between student perceptions of whether the GSU BSPH program curricula prepares students for jobs in the public health workforce, (dependent variable), and study abroad, signature experience, and undergraduate research (independent variables). We also looked at the relationship between GSU BSPH program curricula and student's preparation for the degree program. In the bivariate logistic regression model those who agreed that the GSU BSPH curricula prepares students for graduate education also firmly attested that the GSU BSPH curricula prepares students for jobs. (Unadjusted OR: 8.96, *p*-value = 0.001) (Table 3). Increased odds for preparedness for jobs was observed for all HIPs variables with undergraduate research program being the most significant (Table 3).

**Table 3:** Bivariate analysis of GSU BSPH curricula, study abroad, signature experience, and undergraduate research v. prepares students for jobs.

**Variables**	**Odds ratio**	***P*-value**	**95% Confidence interval**
**GSU BSPH program curricula**			
Would you say that the GSU BSPH program curriculum prepares students for graduate education? Yes/No	8.96	0.001	2.58–31.08
**Study abroad**			
Would you say that GSU study abroad programs are designed in a way that helps students to develop global fluency skills for future careers in public health? Yes/Have not participated	1.31	0.350	0.74–2.33
**Signature experience**			
Would you say that the GSU signature experience course is designed in a way that helps students to demonstrate behaviors that will help them perform well in real-world job situations? Yes/Have not taken the course	1.40	0.316	0.73–2.68
Would you say that the GSU signature experience course prepares students for job interviews? Yes/I do not know	2.92	0.205	0.56–15.35
Would you say that the GSU signature experience course teaches students how to articulate the knowledge and skills they have acquired from the program? Yes/I do not know	1.29	0.411	0.70–2.38
**Undergraduate research**			
Would you say that the GSU BSPH program provides students with research opportunities? Yes/ I do not know	4.39	0.018	1.29–14.99
Would you say that participating in undergraduate research is relevant for professional advancement? Yes/ I do not know	6.72	0.055	0.96–47.07
**Demographics**			
Age, 18–24 yrs vs. 25+	0.57	0.359	0.17–1.89
Academic status: junior to senior	1.12	0.734	0.58–2.15
Gender: male vs. female	0.58	0.402	0.16–2.07
Race: African American vs. all other races	0.90	0.406	0.70–1.16

In the adjusted logistic regression model, when controlling for perceptions on study abroad, signature experience, undergraduate research curricula, gender, academic status, age, and race, we found that the odds that the GSU BSPH curricula prepares students for jobs among students who said the GSU BSPH curricula prepares students for graduate education increased (AOR 30.83, *p*-value 0.001) in relation to their counterparts (Table 1). In this model, students who said that the GSU BSPH program curricula provides them with research opportunities also showed an increased likelihood that the program prepared them for a job in comparison to their counterparts (AOR 6.76, *p*-value = 0.035 (Table 1). A non-significant increased adjusted odds ratio was also observed for study abroad (AOR 1.71, *p*-value = 0.242), as well as higher academic status (AOR 1.20, *p*-value = 0.735) in relation to preparedness for the job market (Table 1).

African American students were less likely to say that the GSU BSPH program curricula prepares students for jobs compared to all other race, although this was not significant (AOR =0.90, *p*-value = 0.615). Males showed a decreased likelihood of preparedness for jobs compared to females; although this was also not significant (AOR =0.27, *p*-value = 0.235) (Table 1).

## Discussion

The study was conducted to assess student perceptions of the role of high impact learning practices such as study abroad, signature experience, and undergraduate research curricula, in preparing students for careers in public health. Findings from the study provide information on whether students felt that the HIPs associated with GSU BSPH program curricula adequately prepares them for careers in public health. The findings also provide a baseline for future assessments.

### GSU BSPH Program Curricula

The GSU BSPH degree program has a generalist curriculum that includes elements of the life and biological sciences, social sciences, and humanities to provide students with an understanding of public health from a broad spectrum of approaches. Students enrolled in the program acquire the knowledge and skills needed to excel in a wide array of public health professions, or to pursue graduate education in the medical, science, social science, or public health fields. Prior to the study, we were convinced that the program's curricula did provide students with a solid foundation to immediately enter the public health workforce and for graduate education. Study results corroborated this conviction as 61.3 and 68.9% of students, respectively indicated that the BSPH curricula does prepare students for jobs and graduate education. This was also affirmed in the multivariate regression model. The GSU BSPH curricula achieves its aims in preparing students for the job market.

### Study Abroad

The Brazil, Dominican Republic, China, Ghana, India, and Uganda are the six countries where students enrolled in the BSPH program have had the opportunity to visit and explore different cultures and life experiences. These programs are typically offered in the summer semester and are led by full-time faculty from the SPH, as well as by faculty from other departments at GSU - geosciences, criminal justice, and communications. Students who participated in these programs indicated in course evaluations that, studying abroad gave them the opportunity to acquire skills such as analyzing public health issues (13.9%), critical thinking (18.1%), problem solving (12.5%), communication of public health issues (13.9%), leadership (12.5%), and intercultural fluency (33%) that they need for their future careers in public health. This is consistent with Gonyea's discovery that studying abroad has a positive impact on student development (9), as well as Lander & Malnarich's observation that studying abroad fosters transformational learning experiences and the development of global citizenship (10).

### Signature Experience

Seniors in the GSU BSPH program complete the signature experience course as part of their culminating experience. This course is implemented in two sections - PHPH/PHPB 4991 and PHPH/PHPB 4992. PHPH/PHPB 4991 serves as the prospectus (planning) course for an experiential, hands on- project that is executed in PHPH/PHPB 4992, the capstone course (11). Both courses are offered every semester in a 7-week mini-mester and can be taken in a single semester. The prospectus course (4991), the first of the two-course sequence, is required to meet the area H requirements (Public Health Signature Experience) of the BSPH program of study. With this section, students have the opportunity to integrate, synthesize and apply their public health knowledge through cumulative and experiential activities designed to assess student acquisition of the required public health competencies covered within the public health major (11).

Students have the option to execute the signature experience course sections in a variety of settings including the classroom, global/study abroad courses, and community- based service projects (11). Both course sections utilize HIPs that are highly engaging and relevant to the coursework that students have completed up to this point in their program of study. Some of the active learning practices include opportunities for students to engage in undergraduate research, study abroad programs, collaborative assignments, integrative projects, and writing intensive assignments (11).

Among the students who said they had participated in the signature experience course, 26.8% said the course helped them to demonstrate behaviors needed for future careers in public health, 13.9% said it helped them to think critically, 18.1% said they were able to acquire interpersonal skills, 12.5% said they learned how to problem solve, 12.5% said the acquired leadership skills, and 18.2% said they gained the ability to communicate public health issues. 18.2% of the students said the course prepared them for job interviews and 30.4% said it helped them to articulate the knowledge and skills they acquired from the GSU BSPH program. According to Aboagye (12), signature experiences are designed to help students think critically, appreciate diversity, and live successfully in a complex rapidly changing world.

### Undergraduate Research

While not all students enrolled in the GSU BSPH program indicated that the program provides them with research opportunities, the majority (71.4%) responded in the affirmative. Most students (89.3%) saw the relevance of undergraduate research for professional advancement. This is consistent with Lopatto's findings that the undergraduate research experiences affect student career plans (13). Dr. Armstrong-Mensah, the lead author of this paper founded and launched the GSU SPH Research and Publication Club in 2018 in response to persistent student requests to do research with faculty. The goal of the club is to build and improve upon the research and writing skills of undergraduate and graduate students, and to give them the opportunity to co-author peer-reviewed publications prior to graduation. In 2018, 25 students signed up with the club's first cohort. To date, 69 BSPH and Master of Public Health (MPH) students have participated in the club and written 23 manuscripts – 11 of these manuscripts have been published, three have just been accepted and are in print, and journal editors just provided comments for two more submissions. The remaining seven manuscripts are in the works to be submitted to peer reviewed journals by the end of fall 2021.

Mentored research within the GSU BSPH program is helping students to think analytically, question critically, be persistent, and to discover the steps involved scientific inquiry. In their study, Nagda et al. found that participating in undergraduate research opened career pathways for students (14) and Kremer et al. indicated that such opportunities led to undergraduate student pursuit of higher education prior to entry into the workforce (15).

### Study Limitations

While study results show that the utilization of HIPs in the GSU BSPH program prepares students for the public health workforce and even graduate education, many of the students had not participated in study abroad (66.7%) and signature experience (73.2) courses. To address this challenge, future studies on this issue will focus only those undergraduate students who have participated in all the HIPs focused on in the study. It must be noted that all GSU BSPH students take the signature experience course toward the end of their senior year, and thus do not miss out on this opportunity. While studying abroad is an effective HIP, it is an optional course at GSU as with most universities because, not all students have the financial means to pay for an international trip even after receiving some assistance in the form of a scholarship. In addition, in as much as some students would like to study abroad, they may not have any elective or an academic course left in their academic pathway that they can use and which would allow them to use FAFSA funds to pay for tuition for the course. This notwithstanding, students benefit from other HIPs in the curriculum that also prepares them for the public health workforce.

## Conclusion

The study focused on three HIPs (study abroad, signature experience, and undergraduate research) utilized in the GSU BSPH program curricula and assessed student perceptions of the role those HIPs play in preparing students for careers in public health. Results from the study showed that the perceived value of HIPs associated with the GSU BSPH program curricula contribute to the preparation of undergraduate students to enter the public health workforce as well as for graduate education. Our sample size and percent of students who had participated in the HIPs was small and thus, prevents us from concretely concluding that the HIPs used in the GSU BSPH curricula prepares undergraduate students to enter the public health workforce. The results provide baseline data for future assessments.

## Data Availability Statement

The original contributions presented in the study are included in the article/supplementary files, further inquiries can be directed to the corresponding author/s.

## Ethics Statement

The studies involving human participants were reviewed and approved by Georgia State University Institutional Review Board. The patients/participants provided their written informed consent to participate in this study.

## Author Contributions

EAA-M wrote the draft manuscript and finalized the manuscript. EAA-M and KR-W edited the final manuscript. EA-M and BY performed the statistical analysis. All authors reviewed and approved the final manuscript.

## Conflict of Interest

The authors declare that the research was conducted in the absence of any commercial or financial relationships that could be construed as a potential conflict of interest.

## Publisher's Note

All claims expressed in this article are solely those of the authors and do not necessarily represent those of their affiliated organizations, or those of the publisher, the editors and the reviewers. Any product that may be evaluated in this article, or claim that may be made by its manufacturer, is not guaranteed or endorsed by the publisher.
